# *Streptomyces coeruleorubidus* as a potential biocontrol agent for Newcastle disease virus

**DOI:** 10.1186/s12917-022-03349-7

**Published:** 2022-06-24

**Authors:** Rewan Abdelaziz, Yasmine H. Tartor, Ahmed B. Barakat, Gamal EL-Didamony, Hanaa A. El-Samadony, Shimaa A. Amer, Marwa M. Gado

**Affiliations:** 1 Department of Microbiology, Faculty of Science, Ain Shams University, Cairo, 11566 Egypt; 2 Department of Microbiology, Faculty of Veterinary Medicine, Zagazig University, Zagazig, 44511 Egypt; 3 Department of Botany and Microbiology, Faculty of Science, Zagazig University, Zagazig, 44519 Egypt; 4 Department of Poultry, Dokki, Agriculture Research Center, Animal Health Research Institute, Giza, 44511 Egypt; 5 Department of Nutrition & Clinical Nutrition, Faculty of Veterinary Medicine, Zagazig University, Zagazig, 44511 Egypt

**Keywords:** *Streptomyces coeruleorubidus*, Telomycin, Antiviral, Hemagglutination activity, Histopathology

## Abstract

**Background:**

Newcastle disease virus (NDV) is a severe disease that affects domestic and wild birds. Controlled antibiotics derived from probiotics have been examined as prospective solutions for preserving seroconversion in NDV-vaccinated fowl. In this study, the secondary metabolite “telomycin” was extracted from *Streptomyces coeruleorubidus* (*S*. *coeruleorubidus*) isolated from Egypt's cultivated soil. The structure of telomycin was determined by the elucidation of spectroscopic analysis, including nuclear magnetic resonance (NMR) and mass spectrometry (MS) spectra, and comparison with the literature. The antiviral activity of the secondary metabolite was tested by checking its effect on NDV hemagglutination activity (HA). Moreover, HA of NDV was tested after inoculation of NDV (control) and a combination of telomycin and NDV in 10- days- specific pathogen-free embryonated chicken eggs (SPF-ECE) daily candling. Histopathological examination was performed for chorioallantoic membranes and liver of SPF-ECE.

**Results:**

*S*. *coeruleorubidus* secondary metabolite “telomycin” showed complete hemagglutination inhibition (HI) activity of NDV strain (MN635617) with log10^6^ infectivity titers (EID50/mL). The HA of NDV strain was 8 log^2^ and 9 log^2^ with 0.5% and 0.75% of chicken RBCs, respectively. Preserved structures of chorioallantoic-membranes (CAM) with dilated capillary networks were observed in the treated group inoculated with telomycin and NDV. Histological changes in SPF-ECE liver were examined after inoculation *in ova* to further characterize the telomycin effect. Telomycin and NDV mixture inoculated group showed preserved cytoarchitecture of hepatocytes with the presence of perivascular foci of lymphocytes. The group that was inoculated with telomycin alone showed normal histology of hepatic acini, central veins, and portal triads.

**Conclusion:**

*S*. *coeruleorubidus* telomycin is a promising bioactive agent that might be a biological weapon against a deadly chicken NDV that costs farmers a lot of money.

**Supplementary Information:**

The online version contains supplementary material available at 10.1186/s12917-022-03349-7.

## Background

The poultry industry represents about 25% of the national income of total meat production [1, 2]. Newcastle disease is now considered a significant problem in chicken production worldwide [1]. Despite the implementation of several vaccination regimens, the Egyptian poultry sector has sustained serious economic losses since the identification of Velogenic Newcastle disease virus (vNDV) genotype VIId [3]. NDV is a bird pathogen that was recently classified into the Genus Avian Orthoavulavirus, family *Paramyxoviridae,* and is now known as Avian Orthoavulavirus-1 (AOAV-1) [4]. NDV has fatal structures due to six genes coding for seven structural proteins: the matrix (M gene), the fusion (F gene), the hemagglutinin-neuraminidase (HN gene), the RNA-dependent RNA polymerase (L gene), the nucleoprotein (NP gene), and the phosphoprotein (p) [5].

Pharmaceutical companies are facing the necessity of producing new drugs. Therefore, the development of a new antiviral agent is critical to finding a natural component with a suitable advantage with a practical pharmacological application in the treatment of infections [5]. These compounds are valuable from industrial, biotechnological, and pharmacological perspectives [1]. Recently, attention has turned toward novel *Streptomyces* species [6] that have an important biological resource for the exploration and discovery of antivirals against different viral infections, including Zika virus, NDV, and influenza A and B viruses [7, 8].

*S. coeruleorubidus* is a bacterium species from the Streptomyces genus that has been isolated from soil [9] and can produce a bioactive compound, which increases the number of potent, unique, modified, unprecedented bioactive secondary metabolites of Actinobacteria [10]. The complex natures of these compounds enable them to be used as potential therapeutic agents to treat the emerging challenging disease. For example, natural cyclopeptides have critical biological activities, such as antitumor, antiviral, antifungal, antibacterial, and antihyperglycemic activities [11].

Telomycin is a secondary metabolite produced by *S. coeruleorubidus;* its structure consists of a cyclic depsipeptide with an attached linear peptide chain (sometimes called a tail). These peptides are called lariat peptides or lasso targets fatal peptides [12]. Also, Johnston et al. [13] supposed that telomycin represents a group of glycopeptides used as antiviral and cancer drugs. It was discovered in 1958, and the structure was subsequently solved in 1968 [14]. Due to its functional groups found in the structure, it has activity against viral systems such as hemagglutinin-neuraminidase (HN) [15]. The secondary metabolites of *S*. *coeruleorubidus* (telomycin: oxaoctaazacyclooctacosine) were studied by investigating its antiviral against influenza virus activity [18].

The resistance of NDV to antiviral agents focuses on three pandemics from the twentieth century, possibly making a sound after time [16]. Because of this virus’s antigenic variations, many studies are carried out during infections to use suitable antiviral [17]. Hence, this study concerns the investigation of antiviral metabolites isolated from Actinobacteria.

## Results

### Characterization of *S. coeruleorubidus* secondary metabolite

**Fig. 1:**
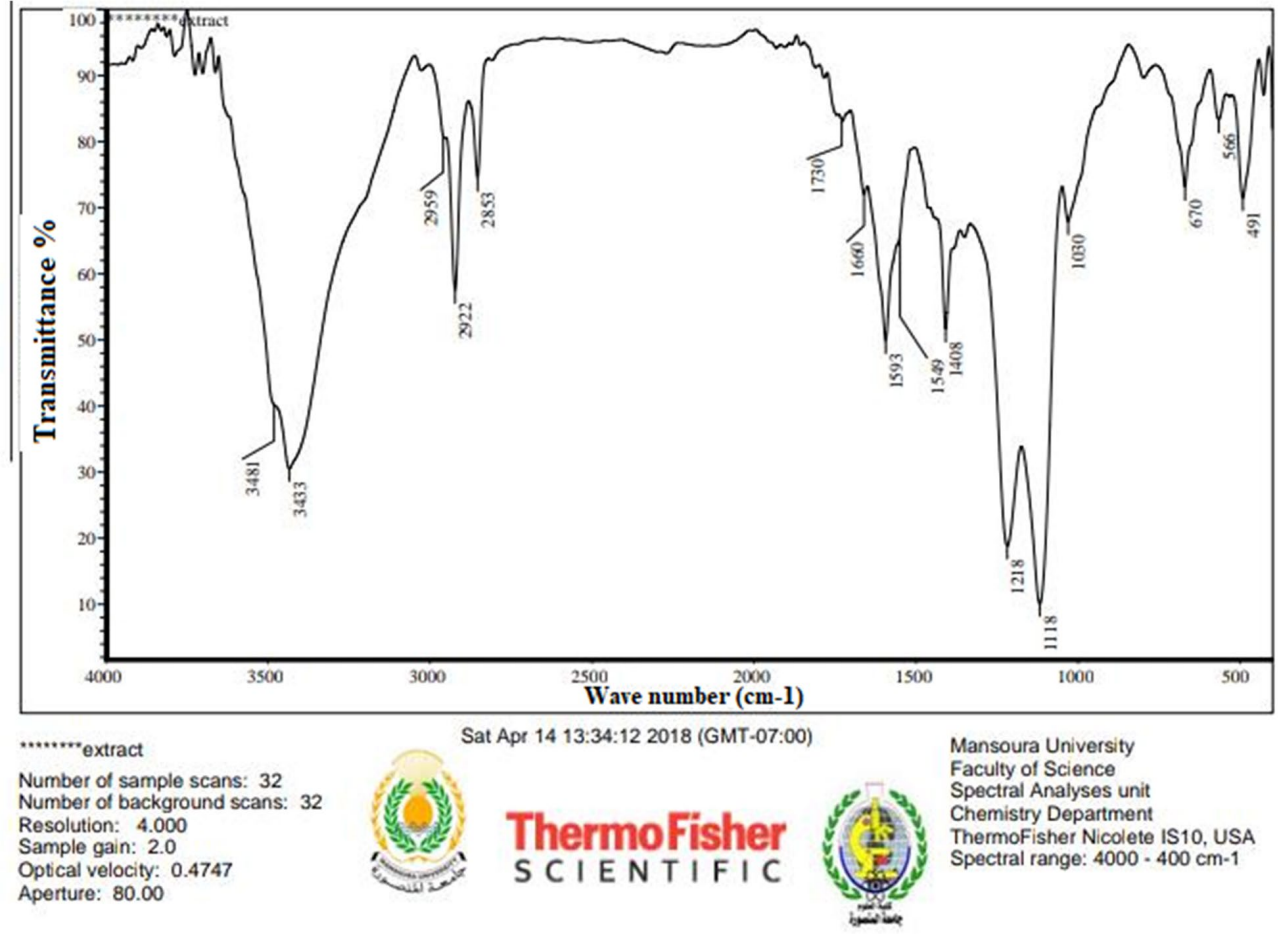
IR spectrum peak report of the antibiotic telomycin using diethyl ether as a solvent

**Fig. 2:**
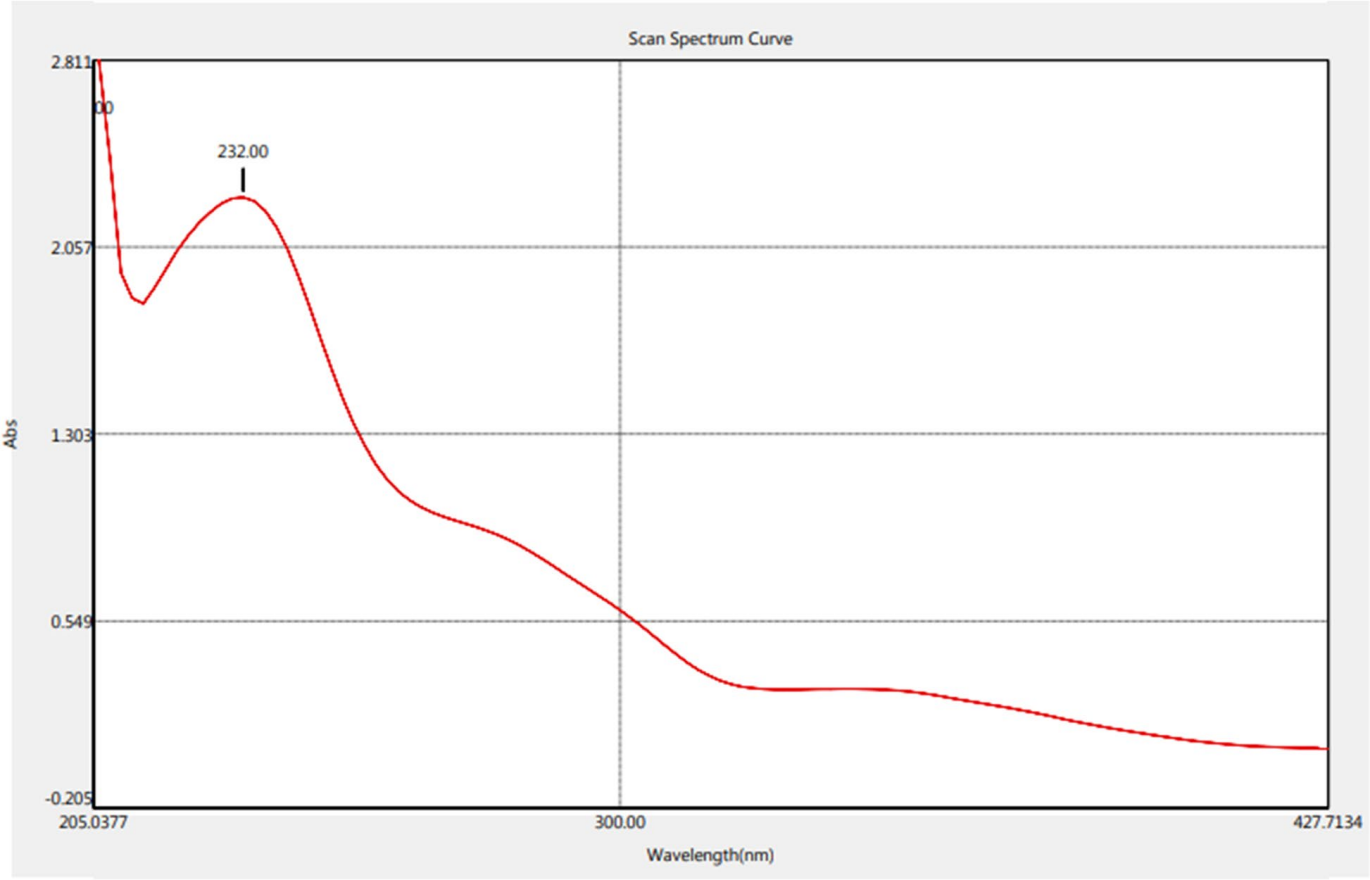
UV spectrum peak report of the antibiotic telomycin using diethyl ether as solvent

**Fig. 3:**
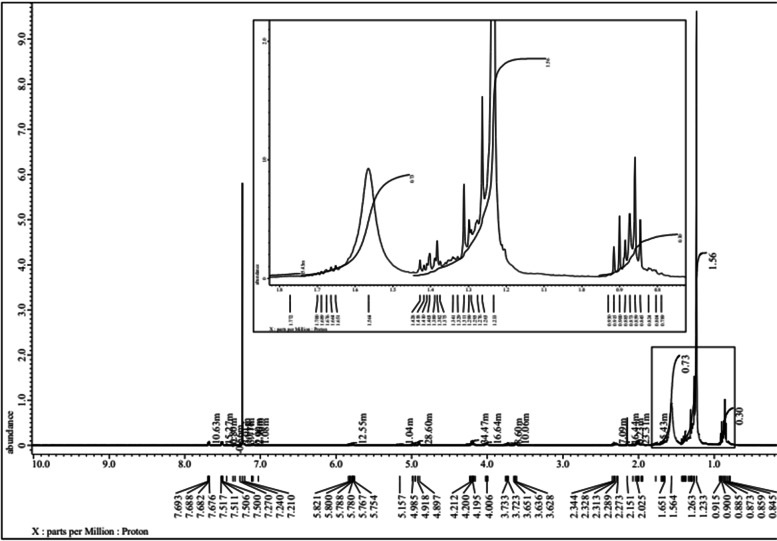
1H1 Peak analysis by NMR spectrum of telomycin antibiotic using chloroform as a solvent

**Fig. 4:**
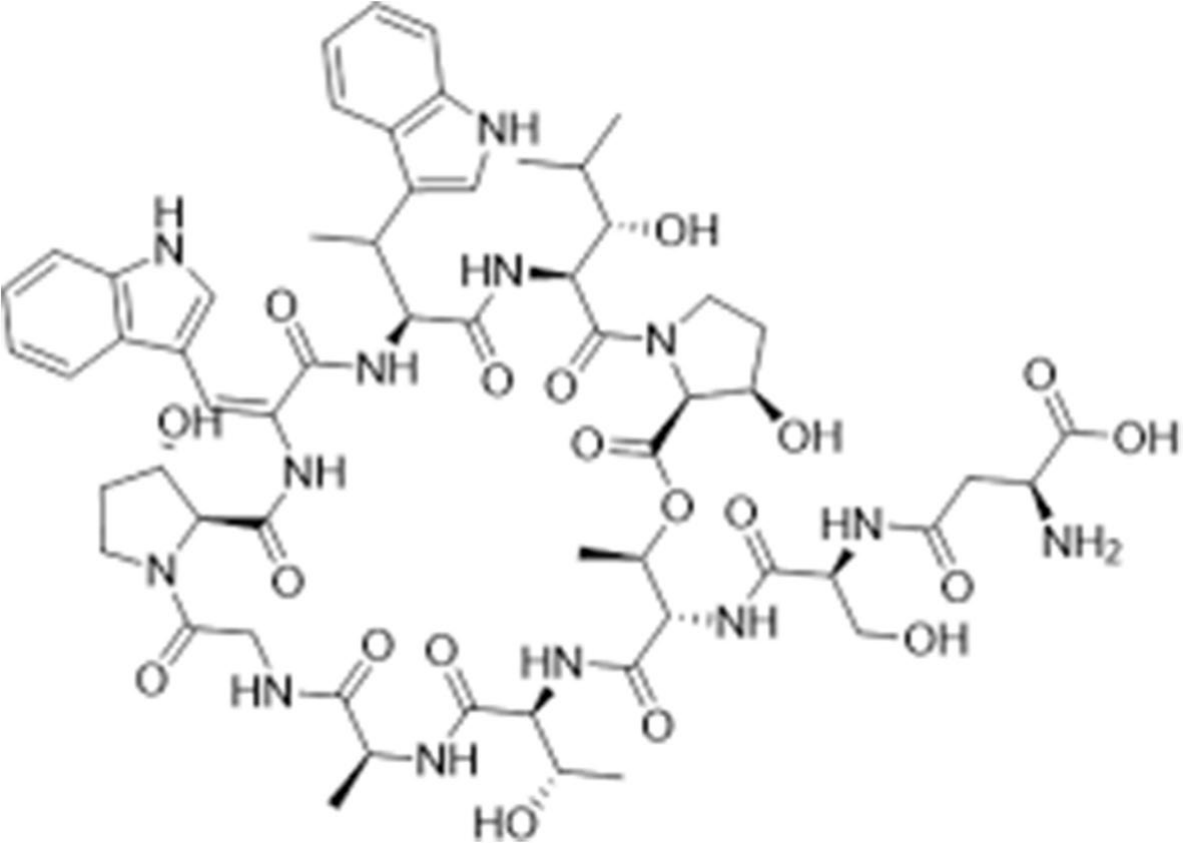
Chemical structure of telomycin as reported by Gurovic et al. [15]

The structure compounds and functional groups of telomycin metabolite were well clarified by chemo-physical instruments, including NMR, IR, and UV. IR spectrum of telomycin displayed absorption spectra at 3433, 2959, 1730, 3481, and 1593 for (Ʋ OH), (Ʋ CH aliphatic), (Ʋ C $=$ O), (amide), and (Ʋ C $=$ C), respectively (Fig. 1). UV spectrum showed the presence of the conjugated system and N $\to\uppi$* transition at 232.00 nm (Fig. 2). Also, C^13^ NMR indicated that the telomycin gave signals at 170.0, 140.0, 114.056, 132.418, 130.892, and 128.793 for SP2 C $=$ O, SP2 C $=$ N, SP2 aromatic carbon, olefinic, SP2 of the aromatic system, and the last three aliphatic SP3 carbon, respectively (Fig. 3). The ^1^H_1_ NMR spectrum revealed$\updelta$, ppm: 0.8 – 0.9 (m, aliphatic, J = 7.5 Hz), 1.2–1.4 (m, O$=\mathrm{H}$, J = 97.5 Hz), 2.0–2.1(m, O$=\mathrm{H}$, J = 35.5 Hz), 7.02, 7.04–7.11(m, NH,s), 7.21–7.27 (m, ArH,s) 7.33–7.36 (m, ArH,s), 7.44–7.50 (m, ArH,s). Analysis of this compound by NMR indicates that this antibiotic is mostly telomycin (Fig. 4).

### Virus titration results in 10-days-old-SPF-ECE

Local NDV strain (MN635617) was propagated in 9–10 day old specific pathogen-free embryonated chicken eggs (SPF-ECE) for 12 passages; allantoic fluids were harvested and tested for sterility. The virus was titrated using infectivity titration on SPF eggs, and the virus titer (EID50/mL) was log10^6^.

### Hemagglutination activity evaluation

To determine the influence of telomycin on the attachment of NDV to chicken erythrocytes, the erythrocytes were pre-treated separately with telomycin. The HA test was then used to assess agglutination using telomycin pre-treated NDV strain (MN635617). As shown in Additional Fig. 1. chicken red blood cells (RBCs) were not agglutinated, which indicates that telomycin can prevent the agglutination of chicken erythrocytes.

The HA of the NDV strain was 8 log^2^ and 9 log^2^ with 0.5% and 0.75% RBCs, respectively. NDV strain and telomycin mixture showed complete inhibition of NDV HA (zero) with chicken RBCs of 0.5% and 0.75% in vitro (Table 1).

**Table 1:** Hemagglutination activity reading of NDV combined with telomycin before and after SPF-ECE inoculation

Treatment with telomycin	RBCs concentration (%)	Telomycin and NDV concentration in PBS			
		**V/V**	**1/2**	**1/4**	**1/8**
Before SPF-ECE inoculation	0.5	zero	zero	zero	zero
	0.75	zero	zero	zero	zero
After SPF-ECE inoculation	0.5	5	4	3	2
	0.75	6	5	4	3

*PBS* Phosphate-buffered saline, *SPF-ECE* Specific pathogen free-embryonated chicken egg

HA of original strain 8 log^2^ and 9 log^2^ with RBCs 0.5% and 0.75%, respectively

The *P-*value of NDV after being treated with telomycin was 0.093 (before inoculation), each at both RBCs concentrations (0.5% and 0.75%). The allantoic fluid was collected after SPF-ECE inoculation with NDV and treated with telomycin (in vivo, SPF-ESE inoculation)

### Hemagglutination activity of telomycin metabolite after inoculation in SPF-ECE

Administration of metabolites in vivo has become a common practice for measuring their efficacy. A virus with telomycin was prepared in different concentrations with phosphate buffer saline and inoculated in SPF-ECE, then measured the potency of telomycin as an antivirus by hemagglutination activity on chicken RBCs. The HA titers (after the virus was treated with telomycin and inoculated in SPF-ECE) ranged from 2 up to 6 log^2^. The virus with telomycin in vitro has completely inhibited HA. After egg inoculation, virus haemagglutination activity returned, but there was a decrease (three logs) from the original strain activity before treatment of the virus. The HA of the original strain (the allantoic fluid) was 8 log^2^ and 9 log^2^ with RBCs chicken 0.5% and 0.75%, respectively, while the *P-*value of NDV after being treated with Telomycin and before egg inoculation was 0.093 each at both RBCs concentrations (0.5% and 0.75%). The (*P-*value = 0.059) after egg inoculation with no significant difference between RBCs concentrations (0.5% and 0.75%) as shown in Additional Fig. 1.

### Histopathological changes in chorioallantoic-membranes and ECE liver

**Fig. 5:**
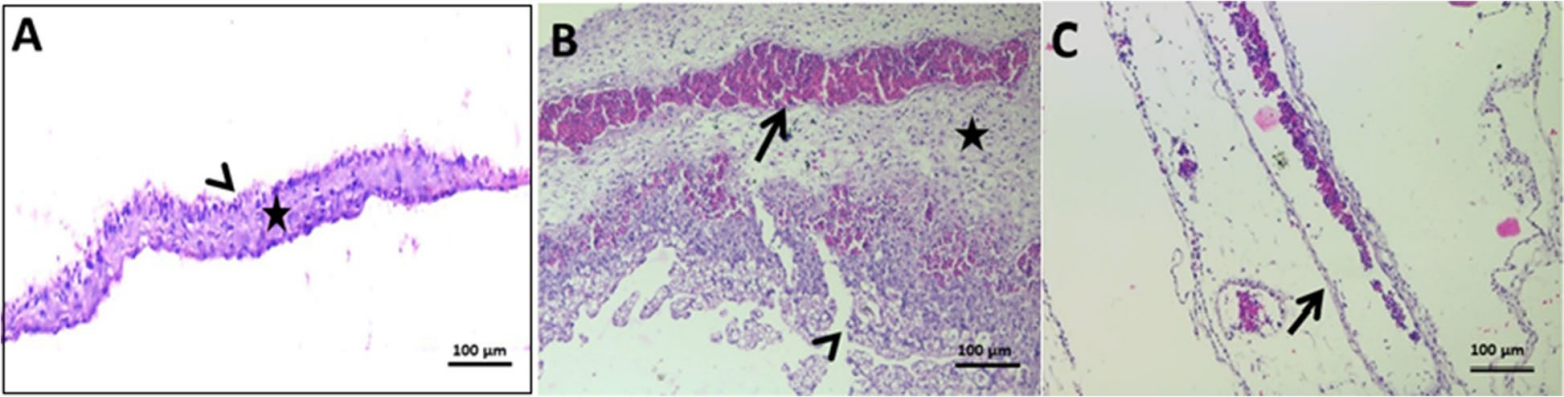
Histological examination of the chorioallantoic membrane after allantoic inoculation of NDV and telomycin mixture in SPF-ECE. **A**: normal chorionic and allantoic epithelium (arrowhead) with a visible stromal region (star) in telomycin group. **B**: hyperplastic and vacuolated chorioallantoic epithelium (arrowhead) and increased thickness of connective tissue layer (star) with numerous hyperemic blood vessels (arrow) in the positive control group. **C**: preserved structures of CAM with the presence of dilated capillary networks (arrow) in NDV and telomycin treated group (H&E X200)

One of the most frequent findings in pathology after inoculating the embryonated egg with a telomycin at various concentrations (in phosphate buffer saline) 1/2, 1/4, and 1/8 (telomycin group) showed normal chorionic and allantoic epithelium with a visible stromal region (Fig. 5 A). The positive control group inoculated with NDV strain MN635617 revealed hyperplastic and vacuolated chorioallantoic epithelium. The increased thickness of the connective tissue layer with numerous hyperemic blood vessels (Fig. 5 B). Preserved structures of chorioallantoic-membranes (CAM) with dilated capillary networks were observed in the treated group inoculated with telomycin and NDV (Fig. 5 C).

**Fig. 6:**
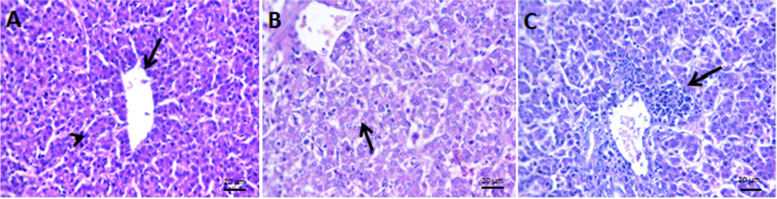
Representative photomicrograph of H&E stained sections of ECE liver with NDV and telomycin mixture by allantoic inoculation harvested in SPF-ECE.**A**: normal histology of hepatic acini (arrowhead) and central vein (arrow) in the healthy group. **B**: vacuolated hepatic parenchyma (arrow) and dilated hepatic blood vessels and sinusoids in the positive control group. **C**: preserved cytoarchitectures of hepatocytes with perivascular foci of lymphocytes (arrow) in NDV and telomycin treated group

To further characterize the telomycin effect, histological liver changes were examined after inoculating in embryonated eggs. The Telomycin group showed normal histology of hepatic acini, central veins, and portal triads (Fig. 6 A). In the positive control group, vacuolated hepatic parenchyma, focal areas of round cell infiltrations, and dilated hepatic blood vessels and sinusoids were observed (Fig. 6 B). However, the treated group showed preserved cytoarchitecture of hepatocytes with perivascular foci of lymphocytes (Fig. 6 C).

## Discussion

Commercially available antiviral drugs do not always succeed, so an urgent problem now needs to be a search for antivirals for several viruses, including influenza, NDV, and paramyxoviruses that are acquiring multi-drug resistance [18]. A potential solution for this emerging issue is to create new antiviral drugs from available compounds of natural products. It is known that the majority of drugs have been developed using compounds derived from Actinobacteria which are naturally occurring Gram-positive bacteria. Examination, isolation, and description of promising strains of Actinobacteria producing possible secondary metabolites have become a significant study concern by many researchers worldwide for many years [19]. Structurally effective and functionally diversified effective compounds have been obtained from Actinobacteria as antibiotics with antibacterial, antifungal, antiparasitic, antiviral, and antitumor activity [3, 4]. Less than one part in 1012 of the earth’s microorganism soil has been screened for Actinobacteria [20]. Telomycin exhibits various bioactivities, including antibacterial, antifungal, antiviral, and antitumor [21]. *S coeruleorubidus* is an industrial microorganism that produces diverse secondary metabolites like telomycin, neomycin, pacidamycin 1, telomycin B1, baumycin B2, baumycin C1, feudomycin A, feudomycin B, feudomycin C, ficellomycin, feudomycinone A, and rubomycin [22]. Isolation of a new Actinobacteria strain, *S. coeruleorubidus,* from a soil sample collected from Zagazig City, Sharkia, Egypt, that produced a complex of new antiviral antibiotics agreed with the findings of Bundle et al*.* [23] who developed an efficient fermentation process for producing secondary metabolites from *Streptomyces* species by adjusting several cultivation parameters like pH, incubation period, and temperature. They reported that 1000 mg/mL of telomycin recorded 90.5% cytotoxicity of Vero cell lines.

The telomycin structural studies suggested a novel type of molecules unrelated to any known antibiotics. The structure component of telomycin have antiviral activity against NDV due to compound fuctional groups [24]. The NMR, IR, and UV analysis results revealed that the composition of the substance was identical to those of telomycin. These results are similar to those obtained by Joo [21] who approve telomycin structure by biochemical instrument.

Structurally telomycin is part of cyclic depsipeptides made up of the amino acids serine, allo-threonine, threonine, alanine, glycine, dehydrotryptophan, β-methyltryptophan, 3-hydroxyproline and 3- hydroxyleucine and often aspartic acid [25]. Giving the ability of telomycin to potently inhibit viral disease due to its structure that contains conjugated double bonds, hydroxyl group, carbonyl group, and an amide group, firstly by partial inhibition of the glycoprotein-C dependent binding of the virions, and secondly by inhibition of events that occur after binding the virus to cells [26]. Another effective drug is doxycycline, which, when combined in a low dose with monocaprin, offers an effective treatment for herpes labialis, significantly reducing the time to healing and pain [26, 27].

The secondary metabolite of *S. coeruleorubidus* (telomycin) has a tremendous potential antiviral effect on the HA of NDV strain (MN635617) with 0.5% and 0.75% of chicken RBCs. The results showed complete inhibition of HA in vitro but decree in these criteria reading three logs than the original in vivo. Therefore, it can be deduced that HA plays a significant role in viral entry, as indicated by numerous studies [28, 29]. This substance (telomycin) affected NDV HA, which targets fusion cells while exhibiting apparent inhibitory effects on the fusion of virus-host cell membrane. This interferes with the function of the hemagglutinin-neuraminidase (HN gene) and the receptor, so there is inhibitory action on the virus's binding activity and fusion activity. HA inhibition (HI) would result in the interruption of virus entry to the infected host cells, leading to prophylactic and therapeutic effects toward NDV infection [18].

In the negative control SPF-ECE groups (telomycin only), non-significant histological changes were observed in the CAM and embryo liver. Histological changes in NDV inoculated embryos ranged from congestion, bleeding, and hyperplasia with different degrees in CAM layers and embryo liver. The data showed that telomycin possesses a broad and potent anti-NDV activity in vitro while in vivo exhibits a different mode of action and may need more studies to reach new antivirals activity in vivo effect.

## Conclusions

The findings concluded that *S. coeruleorubidus* creates a secondary metabolite called "Telomycin," which operates as a biocontrol agent for the NDV. Telomycin showed an antiviral effect in vitro plus no lethal effect on SPF- ECE at different concentrations, while in vivo exhibits preserved cytoarchitecture of hepatocytes with the presence of perivascular foci of lymphocytes.

## Methods

The experimental protocol was approved by the Ethics of the Institutional Animal Care and Use Committee of Zagazig University, Egypt (ZUIACUC–2019). All animal experiments were performed following recommendations described in “The Guide for the Care and Use of Laboratory Animals in scientific investigations."

### Cultivation of *S. coeruleorubidus* and metabolite purification

To investigate the antiviral characteristics of *S. coeruleorubidus* secondary metabolites, the *S. coeruleorubidus* strain was grown in starch nitrate broth (pH 7.2) containing starch (10 g), CaCO3 (3 g), MgSO4 (0.5 g), K2HPO4 (1 g), NaNO3 (2 g), and NaCl (5 g) per liter (Oxoid, UK). After 5 days of incubation at 28 °C in a 100 mL total culture, the broth from 2 different conical flasks was combined (2 flasks were cultured per strain) and separated from the mycelium by filtration through a coarse piece of clothing using Büchner porcelain funnels (Stonylab Egypt o prepare cell-free culture broth. The residue of the *S. coeruleorubidus* cells was discrete by centrifugation at 13,000 rpm for 15 min. The metabolite solution gained after filtration and centrifugation was extracted using N-butanol (in a ratio of 5:1). Extraction was further completed in the refrigerator at 4 °C for a day. The N-butanol layer was separated using a separator funnel and filtered through a filter paper. The collected N-butanol fraction was concentrated in a vacuum on a rotary evaporator (RV-10 Basic, stonylab Egypt) to a dry residue. The obtained compound was dissolved in 3 mL ethanol (80%) and stored at 4 °C until further analysis [24].

### Chemicals and structural elucidation for Telomycin

The chemical properties of *S. coeruleorubidus* metabolite were studied using ultraviolet spectrum (UV), infrared spectrum (IR), Nuclear magnetic radiation spectrum (NMR), and chemical shifts were referenced to tetramethylsilane (TMS Oxoid, UK) as an internal standard at the Faculty of Science, Zagazig University.

### Thin-layer chromatography

The thin layer chromatography (Sigma, St. Louis, MO) for fractionating the obtained metabolite was done using pre-coated TLC sheets to detect antibiotics. The obtained fraction was dissolved in diethyl ether for assaying the bioactivity using the disc diffusion method [30]**.**

### UV Spectrum

The ultraviolet spectrum of the antibiotic was scanned on a UV spectrophotometer (UV–Vis spectrophotometer, Thermo Scientific Multiskan Sky High Microplate Spectrophotometer, Germany). The spectrum was scanned at a wavelength of 200 nm using diethyl ether solution. The absorbance value of each peak analysis was recorded [31].

### Fourier Transform Infrared Spectroscopy (FTIRS)

The infrared spectrum of the substance was scanned on an IR spectrophotometer (ThermoFisher Nicolete IR IS10- USA). The spectrum was scanned between 4000–400 cm-1 using diethyl ether. The spectra were plotted as intensity versus wave number. In this spectrum range, maximum and minimum resolution and the number of the peaks were recorded [32].

### Nuclear magnetic radiation spectrum (ECA-500II) (NMR)

In this spectrum, chloroform was used as a solvent. NMR spectroscopy is mainly an analytical method to obtain detailed structural and quantitative information about the metabolites produced. Include 1D-1 H, 1D 1 H decoupled 13C, 2D 1H J-resolved, 1 H-1 H NOESY, 1 H-1H COSY, 1 H-1 H TOCSY, 1 H-13C HSQC and 1 H-13C HMBC experiments. Data obtained were compared with similar compounds produced by *S. coeruleorubidus* [33, 34].

### In Vivo testing of Streptomyces metabolite preparation

#### Virus identification

NDV (GenBank accession No. MN635617) was isolated from a chicken farm in Dakahlia Governorate, Egypt, in February 2019 following consultation with farm owners who had contacted the Animal health research institute, Dokki, Giza, to discuss their current situation. The identification was confirmed with NDV primers (Forward 5'-TTG ATG GCA GGC CTC TTG C-3’ and Revers, 5'-AGC GTY TCT GTC TCC T-3’ [35].

#### Virus propagation and titration

The NDV strain was titrated on allantoic fluid through inoculation of 0.1 ml of the virus in 10-days-old SPF-ECE (Kom Oshem, SPF Farm, Fayoum, Egypt) with daily candling [36]. According to Reed and Muench [37], allantoic fluid was collected 72 h post-inoculation to calculate infectivity titers as Embryo Infective Dose 50 /mL (EID50).

### Telomycin effect on NDV hemagglutination activity

The specified virus-inhibitory effect of the investigated telomycin was obtained with an equal volume of 100 EID50/mL of the MN635617 NDV tested strain. After 30 min of incubation at 37 °C, the mixture was examined for hemagglutination activity with chicken red blood cells (RBCs) of 0.5% and 0.75%. Phosphate-buffered saline (pH 7.2) was used as a negative control [38].

### Inoculation of SPF-ECE

Red blood cells were obtained from adult healthy chicken (4 cm from one bird) from the Jugular vein of the neck by sterile syringe 1-4 cm on 4% sodium citrate as an anticoagulant, the cells were washed 3 times with normal saline at 3000 rpm and the packed cells were diluted as 10% and 0.5% to be used for rapid and plate haemagglutination (HA) and haemagglutination inhibition (HI) tests in V-bottomed microwell plates according to the OIE Manual of Standard Diagnostic Tests [39]. After SPF-ECE inoculation, the allantoic fluid was evaluated for haemagglutination activity using micro well plates for the HA activity test [36].

SPF eggs inoculation with twofold dilutions of NDV strain and telomycin ( in Phosphate-buffered saline1/2, 1/4, and 1/8) incubated for 30 min at 37 °C [38].

### Inoculation of telomycin and NDV in SPF -ECE

Three dilutions from telomycin (1/2, 1/4, and 1/8) were prepared and incubated at room temperature for 30 min with NDV strain, then inoculated into an allantoic sac of 10 day-old SPF- ECE / 5 eggs/ 0.2 mL each, incubated at 37 °C in an egg incubator and daily observed for deaths. Three days later, the live eggs were placed at 4 °C overnight, and the allantoic fluids were collected to examine NDV hemagglutination activity [40] with 0.5% and 0.75% of chicken RBCs.

### Histopathological examination of SPF-ECE

The collected CAM and liver of the inoculated SPF-ECE were fixed in 10% buffered neutral formalin solution, and paraffin Sects. (5 microns thickness) were prepared and stained with hematoxylin and eosin (H&E) [41]**.**

### Data analysis

The telomycin and NDV strain results on the same RBCS concentration were analyzed using a T-test. *P* ≤ 0.05 were considered statistically significant.

## Supplementary Information

**Additional file 1:** **Fig.1** Mixture of NDV and telomycin for 30 min at roomtemperature then inoculated in ova. After 3days of collected allotonic fluidand examination, A: Represents Hemagglutination assay of harvested allantoicfluid after mixture inoculation NDV (MN635617)in SPF-ECE, which inhibits chicken Red Blood Cells with 00.5%; B: Represents Hemagglutinationassay of harvested allantoic fluid aftermixture inoculation NDV (MN635617) in SPF-ECE inhibition of chickenRed Blood Cells with 0.75 %.

## Data Availability

All data generated or analyzed during this study are included in this article.

## Abbreviations

NMR: Nuclear magnetic resonance
FTIRS: Fourier transform infrared spectroscopy
MS: Mass spectrometry
NDV: Newcastle Disease Virus
HI: Hemagglutination Inhibition
SPF-ECE: Specific Pathogen Free Embryonated Chicken Eggs
HA: Hemagglutination Activity
EID50: Egg Infective Dose
