# Supplementary material for: Streptomyces coeruleorubidus as a potential biocontrol agent for Newcastle disease virus
Source: BMC Vet Res. 2022 Jun 24;18:241. doi: 10.1186/s12917-022-03349-7 (PMC9229119; doi:10.1186/s12917-022-03349-7)
Supplement: Supplementary file 1 — Additional file 1: Fig.1 Mixture of NDV and telomycin for 30 min at roomtemperature then inoculated in ova. After 3days of collected allotonic fluidand examination, A: Represents Hemagglutination assay of harvested allantoicfluid after mixture inoculation NDV (MN635617)in SPF-ECE, which inhibits chicken Red Blood Cells with 00.5%; B: Represents Hemagglutinationassay of harvested allantoic fluid aftermixture inoculation NDV (MN635617) in SPF-ECE inhibition of chickenRed Blood Cells with 0.75 %. [file 12917_2022_3349_MOESM1_ESM.docx]

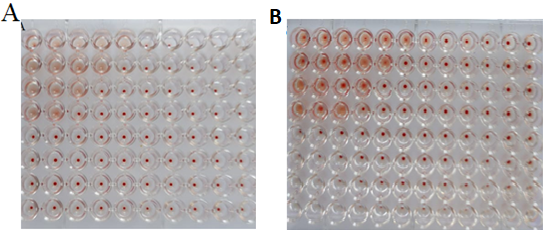


**Additional Fig. 1** mixture of NDV and telomycin for 30 min at room temperature then inoculated in ova. After 3days of collected allotonic fluid and examination, A: Represents Hemagglutination assay of harvested allantoic fluid after mixture inoculation NDV (MN635617) in SPF-ECE, which inhibits chicken Red Blood Cells with 00.5%; B: Represents Hemagglutination assay of harvested allantoic fluid after mixture inoculation NDV (MN635617) in SPF-ECE inhibition of chicken Red Blood Cells with 0.75 %.
